# Migrating leukocytes are the source of Peroxiredoxin V during inflammation in the airways

**DOI:** 10.1186/1476-9255-3-13

**Published:** 2006-10-04

**Authors:** Raisa I Krutilina, Andrei V Kropotov, Christian Leutenegger, Vladimir B Serikov

**Affiliations:** 1 Institute of Cytology Russian Academy of Sciences, St. Petersburg, 194021, Russia; 2 University of California, Davis, Davis, CA 95616, USA; 3 Children's Hospital Oakland Research Institute, Oakland, CA 94609, USA

## Abstract

**Background:**

We characterized changes in expression of the antioxidant protein Peroxiredoxin V (PRXV) during airway inflammation.

**Methods:**

Studies in anesthetized rats and mice; PRXV expression determined by Western blot analyses and immunohistochemistry; PRXV m-RNA expression determined by Taq-Man RT-PCR.

**Results:**

Bacterial lung inflammation did not change expression of PRXV in murine epithelia but produced massive influx of leukocytes highly expressing PRXV. Endotoxin and f-MLP induced leukocyte migration in rat trachea but did not change mRNA levels and PRXV protein expression in tracheal epithelial cells. In primary airway cell culture (cow), alveolar epithelial cells A549, or co-culture of A549 with murine macrophages RAW264.7, exposure to live bacteria increased expression of PRXV, which required serum. PRXV was secreted *in vitro *by epithelial and immune cells.

**Conclusion:**

Inflammation increased expression of PRXV in airways by at least 2 mechanisms: cell population shift by massive influx of leukocytes expressing PRXV, and moderate post-transcriptional up-regulation of PRXV in epithelial cells.

## Background

To ensure adequate protection against oxidative stress during states of pulmonary disease, several antioxidant systems have evolved in the epithelial cells of mammalian airways [1-4]. Peroxiredoxins I-VI (PRX I-VI) are a group of potent antioxidant proteins that are the subject of much research [5-10]. PRXs neutralize reactive oxygen by transferring electrons from thioredoxins or cyclophilins. The six PRXs differ in their intracellular distribution and are thought to serve different functions and be regulated by different mechanisms. PRXV is one of the key enzymes of cellular antioxidant defense, as it is a potent protector against DNA damage and also has other functions [11-14].

Toxic insults to the respiratory tract down-regulate synthesis of the PRXV protein. We have recently demonstrated *in vivo *in rat tracheal epithelial cells that cigarette smoke extract (CSE) directly down-regulated expression of PRXV, which is one mechanism of cigarette smoke toxicity [15]. Exposure of isolated tracheal segment to CSE significantly reduced mRNA levels for PRXV and the amount of PRXV protein in the epithelium. In cultures of the tracheal epithelial cell lines, primary airway cell culture, and the alveolar epithelial cells A549, CSE significantly decreased transepithelial electrical resistance, expression of PRXV protein, and significantly induced glutathione and protein oxidation. Similarly, when respiratory tract toxicity was induced in mice with naphthalene, the loss of the Clara cell population was associated with a significant decrease in PRXV expression [16]. In contrast, previous reports had indicated that PRXV was over-expressed in the lung during inflammation induced by endotoxin [17]. However, experiments *in vitro *in which pro-inflammatory cytokines were added to human alveolar or bronchial epithelial cells did not result in an up-regulated expression of PRXV [18]. Neither the mechanism by which PRXV is up-regulated during inflammation in tissues of the lung nor the identity of the cells that are the source of PRXV production are known.

We therefore investigated the effects of gram-negative bacterial inflammation on expression of PRXV in lung, lung epithelial cells, and immune cells *in vivo *and *in vitro*. Our first aim was to determine whether inflammation *in vivo *influences expression of PRXV in the bronchial epithelium and alveoli. Our second aim was to use an *in vivo *model of inflammation to investigate whether changes of transcription or translation of PRXV in the tracheal epithelium, if they occurred, were a direct response to bacterial pathogen lipopolysaccharide (LPS) by these cells or whether the increased level of PRXV was induced by leukocyte migration. Our third aim was to determine *in vitro *whether exposure of the airway and alveolar epithelial cells to live bacteria, either alone or in co-culture with murine macrophages RAW264.7 changes the level of PRXV mRNA as well as protein expression and secretion.

We found that both *in vivo *and *in vitro *inflammation induced by bacteria resulted in an increased expression of PRXV in the airway epithelium by at least 2 different mechanisms: massive influx of activated leukocytes, which highly express PRXV, and moderate translational up-regulation of PRXV in the epithelial cells.

## Methods

### 1. In vivo studies

Experiments in animals were performed according to protocols approved by the Institutional Animal Use Committee of the Children's Hospital Oakland Research Institute and Institute of Cytology, RAS.

#### Experiments in mice

##### Bone-marrow transplantation

Recipient mice (n = 12) were given a sub-lethal dose of whole-body irradiation (5.05 Gy) the day before transplantation. While under general anesthesia (Pentobarbital, 25 mg/kg IP), the mice were infused with 10^6 ^whole bone-marrow cells in 0.2 ml of PBS into the jugular vein.

##### Bacterial lung injury

In the experimental group, six chimeric mice received intratracheal instillation of PBS (n = 3, control) or 7 × 10^6 ^cfu of *E. coli *K12 JM109 in 50 μl of PBS; the chimeric model has been described previously [16]. As a secondary control group for the bacterial inflammation study, 3 non-chimeric C57BL/6 mice received 7 × 10^6 ^cfu of *E. coli*, while 3 non-chimeric mice without known lung pathology were used as controls. These mice were euthanized and studied 1–2 weeks after the *E. coli *instillation.

#### Experiments in rats

##### Perfusion of rat trachea

An anesthetized Sprague-Dawley rat model of an *in situ *perfusion of isolated tracheal segment with an intact blood supply was used, as described previously [19]. Experimental groups: *Control group *(n = 6): In the control group, tracheal segments were filled with PBS and sampled at 2 and 4 hours thereafter. *Induced leukocyte migration *(n = 4): In this group, 5 × 10^-8 ^M f-MLP (final concentration) was added to tracheal lumen in PBS and samples were taken at 4 hours. *Endotoxin model *(n = 4): In this group, LPS *E. coli *O55:B5 at a concentration of 100 μg/ml was applied to the inner trachea for 4 hours.

At the end of the experiment in all groups, tracheal lumen was thoroughly washed, and samples of the epithelial layer from the tracheas were cut out, frozen in liquid nitrogen, and further used for RT-PCR or immunohistochemical analyses to determine expression of mRNA or PRXV protein.

### 2. In vitro cell culture experiments

Cell culture techniques used have been described previously [20].

A549 (ATCC) cells were grown in Hank's F12 K medium with 2 mM L-glutamine, 10% fetal bovine serum (FCS) (Life Technologies, Gaithersburg, MD), and streptomycin/penicillin. Co-culture experiments were performed in DMEM with or without 10% heat-inactivated FCS. *P. aeruginosa *PAO1 was added for 12–24 hours to the apical surface at a concentration of 5 × 10^7 ^cfu/ml. Following exposure, cells were washed 3 times with PBS and then either fixed with 4% paraformaldehyde for 24 hours for IHC or collected for Western blot analyses in cell lysis buffer on ice. Experiments were performed in triplicate in 3 different cultures.

Bronchial epithelial cells Calu-3 (ATCC) (gift of Dr. T. Machen, University of California, Berkeley) were grown on the internal surface of polycarbonate membranes (0.3 μm pore size, 6.5 mm diameter) in Transwells (Costar, Cambridge, MA) with an air-liquid interface. These cells were similarly exposed to PAO1 for 12 hours at a concentration of 5 × 10^7 ^cfu/ml. TER, a measure of tight junctional permeability, was measured with a voltmeter (EVOMX-G, World Precision Instruments, Sarasota, FL). CTE cells were grown and studied similarly. Primary cultures of cow tracheal epithelial (CTE) cells was performed as follows: Surface of the cow tracheas was scored into thin strips and those were separated from the underlying cartilage rings and placed in cold phosphate buffered saline (PBS) + PSFG (Penicillin, Streptomycin, Fungizone, Gentamycin). Strips were placed in 40 ml of Hank's BSS, Ca^2+^/Mg^2+ ^free + PSFG with 1 mg/ml protease (Sigma Co), and digested overnight at 4°C. Strips were then resuspended in DME H21/F-12 mix + 5% FCS + PSFG, shaken vigorously to pull the cells off. The cell suspension was centrifuged for 10 minutes at 1000 rpm. The cells were plated 10^6 ^cells/cm^2 ^on 3 μ pore polycarbonate membranes and grown in DME-H21/F-12 mix with PSFG and a mixture of growth factors consisting of transferrin, insulin, triiodothyronine, hydrocortisone, endothelial cell growth supplement, and epidermal growth factor. As CTE cells were more resistant to PAO1 than were the Calu-3, exposure to 5 × 10^7 ^cfu/ml of bacteria was extended to 12 hours.

RAW 264.7 (ATCC) were grown in RPMI-1640 with 15% FCS, THP-1 (ATCC) were grown in RPMI-1640 medium with 2 mM L-glutamine adjusted to contain 1.5 g/L sodium bicarbonate, 4.5 g/L glucose, 10 mM HEPES and 1.0 mM sodium pyruvate and supplemented with 0.05 mM 2-mercaptoethanol, 10% FCS.

### 3. Western Blot analyses and immunohistochemistry

These were performed as described previously [20]. *Antibodies used*: Anti-Green Fluorescent Protein rabbit IgG, 1:50, (Molecular Probes, Eugene, OR), rat anti-mouse CD45 antibody 1:10 (Calbiochem San Diego, CA), secondary anti-rat, anti-rabbit antibodies (Molecular Probes,, Eugene, OR). Our own anti-PRXV rabbit antibody [12] at 1:200 dilution was used for PRXV staining.

### 4. Analyses of PRXV mRNA expression

Taq-Man analyses were performed at the University of California, Davis, Lucy Whittier Molecular and Diagnostic Core Facility, at the Department of Medicine and Epidemiology by using standard techniques. For rat PRXV gene, pre-developed TaqMan PCR assay (Rn00586040-m1) was purchased from Applied BioSystems (Foster City, CA). In order to determine the most stably transcribed housekeeping gene, a housekeeping gene validation experiment was conducted on a representative number of samples. The housekeeping gene with the least standard deviation in all treatment groups (HPRT1 or TFR2) was used to normalize the target gene CT values. All gene transcriptions were expressed and are presented here as an n-fold difference relative to the calibrator.

### 5. Statistical analyses

At least six different sections from each lung were used for analyses. Cell counting was performed in 20 different randomly selected visual fields. Numbers of GFP^+ ^cells were determined as a percentage of the total number of cells (counted by numbers of PI-stained nuclei). In antibody-specific staining, numbers of ligand-positive cells were expressed as a percentage of the total numbers of GFP^+ ^cells in 20 different visual fields. Fluorescence intensity in cells was determined by built-in Zeiss LSM software options. Western blot analyses was performed in samples from 3 different cultures; results were quantified by photometry. Data are presented as the MEAN ± SE, statistical significance by ANOVA or Student's *t*-test was established at *p *< 0.05.

## Results

### 1. Migrating leukocytes are the source of PRXV in the lung

**Figure 1:**
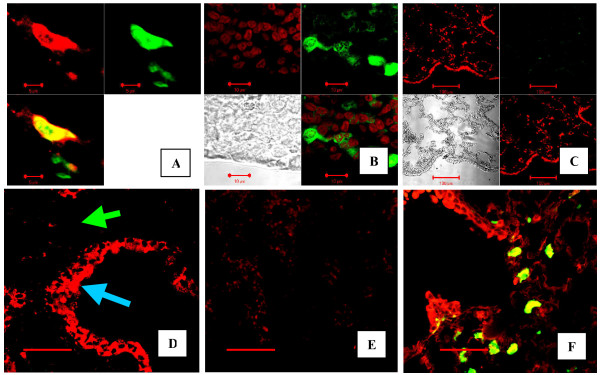
A-C: Bone marrow-derived GFP^+ ^cells infiltrated the lung following acute bacterial pneumonia. Confocal microscopy images of the lung. Irradiated mice were transplanted with whole bone marrow from GFP^+ ^Tg mice. After induction of pneumonia by *E. coli *instillation, lungs were fixed and stained for GFP with anti-GFP antibodies. A: Cryosection of the lung, which shows co-localization of signal from Texas Red-labeled antibody against GFP (red, upper left panel) with GFP signal (green, upper right panel). The lower panel is a combined image. B: Paraffin section of the lung from the same experiment. Lungs are co-stained for DNA with Propidium Iodine (Upper left panel) and stained with anti-GFP antibody and secondary FITC labeled antibody (Upper right panel). Lower left panel – tissue image in reflected light, lower right panel – combined image. C: Control staining of paraffin-sectioned lungs with isotype primary antibody, no non-specific green fluorescence can be noted, same panel description as in B. D-E: PRXV was abundantly present in cells of the bronchial epithelium of mice, and acute bacterial inflammation did not further significantly increase it. Confocal microscopy images of the cryosectioned lung, stained for PRXV with red-fluorescent secondary antibody. D: – Non-inflamed control lung (cryosection), original magnification × 40, bar is 50 microns. Note high expression of PRXV in the bronchial epithelium (blue arrow) but not in the alveoli (green arrow). E: Control staining with isotype primary antibody; no non-specific red fluorescence is present. F: GFP^+ ^cells, which are present in high numbers in the lung following pneumonia, highly express PRXV. Cryosection of the lung, stained for PRXV with Rhodamine-labeled antibodies (red). Fluorescence intensity of the bronchial epithelium does not differ from control (Panel D). Note the presence of bright green GFP^+ ^(or yellow due to superposition of green GFP and red PRXV signals) cells, which also highly express PRXV.

We used a chimeric model to study the presence of leukocytes in the lung during inflammation. Transplanted mice demonstrated 30–50% bone marrow chimerism 3 months after transplantation. Engraftment of GFP^+ ^cells to the lungs of control mice (non-injured lungs) was found to be distinctive, but at a very low level (0.001–0.1%). IHC and confocal microscopy allowed us to readily identify GFP^+ ^cells and determine the expression of PRXV (Figure 1).

Half of the mice subjected to LD_50 _intratracheal instillation of live *E. coli *died from pneumonia within 1 week. In the surviving mice, the peak of lung inflammation (7 days after *E. coli *instillation) was predominantly associated with the influx of GFP^+ ^leukocytes, which represented 16 ± 3 % of total lung cells. 95% of GFP^+ ^cells in the lung were CD45^+ ^cells.

**Figure 2:**
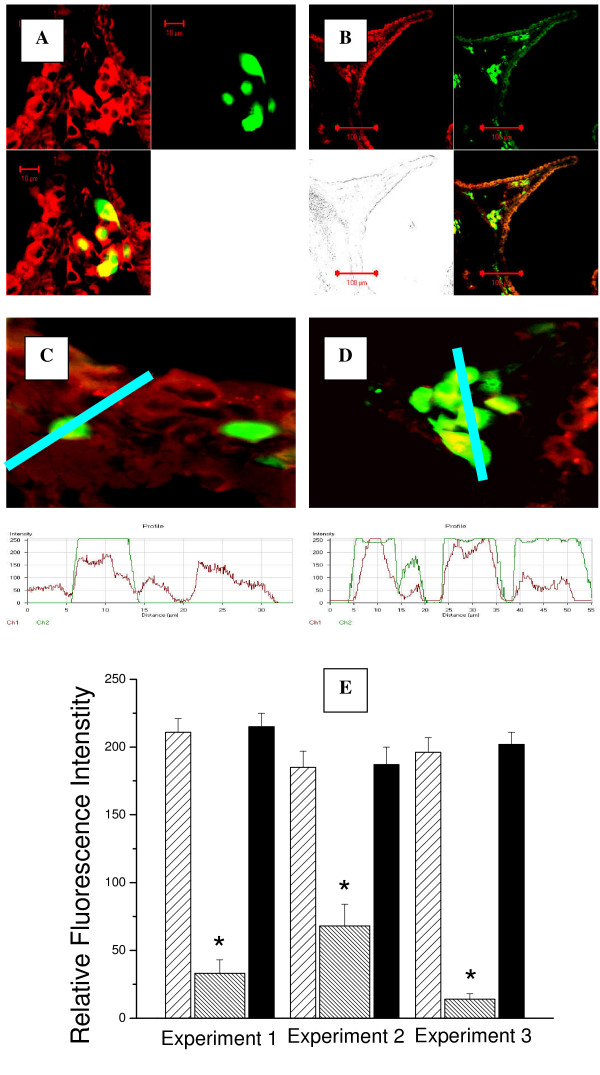
Following bacterial inflammation, GFP^+ ^cells in the lung highly expressed PRXV. Animals were transplanted with GFP^+ ^bone marrow, and progeny of GFP^+ ^cells (green fluorescence) was located to the sites of inflammation in the bronchial epithelium. Confocal microscopy images of the cryosectioned lung stained for PRXV with red fluorescent antibodies. A: GFP^+ ^cells in the peribronchial interstitial spaces following inflammation of the lung, upper left panel – PRXV staining (red fluorescence), upper right panel – GFP fluorescence (green), lower panel – combined image. B: GFP^+ ^cells in the wall of the large bronchus. Upper left panel – PRXV staining (red fluorescence), upper right panel – GFP fluorescence (green), lower left panel – reflected light image, lower right panel – combined image. C-D: Fluorescence intensity of PRXV label (red) co-localized with green GFP signal in the lung tissues. At the bottom of each image the profile diagram of distribution of fluorescence intensity along selected segment (blue bar) is given. Red line is PRXV fluorescence intensity, green line is GFP fluorescence intensity. Original magnification × 40. E: Summary results of relative PRXV fluorescence intensity in the bronchial epithelium (loose shade bar), alveolar walls (dense shade bar), and GFP^+ ^cells (black bar) present in the lung from 3 different experiments. * – *p *< 0.05.

Using this model, we first determined the level of expression of PRXV in the cells of the murine bronchial epithelium (Figures 1 and 2). PRXV was abundantly expressed in the bronchial epithelium of the lungs of control mice. PRXV expression in the bronchial epithelial cells was several-fold higher than in the cells of alveoli. We did not observe significant changes in the level of PRXV expression in the bronchial epithelial cells during acute inflammation (Figure 2). Similarly, we did not observe a significant increase in PRXV expression in the cells of alveolar epithelial lining during inflammation. However, during the development of inflammation, multiple leukocytes appeared in the lung parenchyma, most of which highly expressed PRXV (Figure 2). Therefore, infiltration of the lung parenchyma with leukocytes resulted in an enhanced overall expression of PRXV at sites of inflammation.

### 2. PRXV protein expression is up-regulated in rat tracheal epithelium cells by f-MLP

We then used a perfused tracheal segment *in vivo *rat model to determine whether short-term (4 hours) exposure to f-MLP (induced leukocyte migration) or bacterial (*E. coli*) LPS would enhance transcription and translation of PRXV in the tracheal epithelium. Following exposure to f-MLP or LPS, the tracheal segment was carefully washed off the cells in the lumen. In our previous studies, 4 hours of exposure of tracheal segment to f-MLP resulted in enhanced leukocyte migration and increased permeability [19,20]. We therefore used this time period to assess expression of PRXV in the model of inflammation. In the f-MLP model of inflammation, a 4-hour exposure of the isolated tracheal segment to f-MLP provided a small (32%) yet significant (*p *< 0.05) increase in the PRXV expression in the cells of tracheal epithelium (from 182 ± 16 relative units in the control to 241 ± 3 relative units in the experimental group), but not in mRNA levels (2.36 ± 0.23 in the control *versus *1.51 ± 0.22 in the experimental group). In the LPS model, we also did not observe statistically significant difference in PRXV mRNA levels in the tracheal epithelium (4.71 ± 0.9 in the control *versus *2.3 ± 0.7 in the LPS experiment model). There were no significant differences in PRXV protein expression in the epithelium (data not shown).

### 3. Live P. aeruginosa bacteria up-regulates expression, but not transcription, of PRXV in cultured airway epithelium in the presence of serum

**Figure 3:**
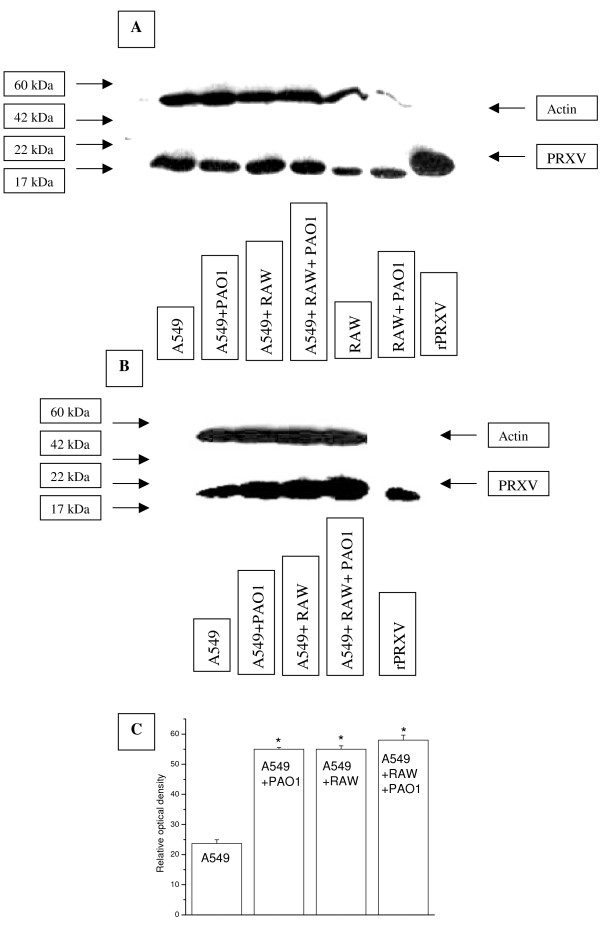
*P. aeruginosa *infection up-regulated expression of PRXV protein in cultures of the A549 epithelial cells, and co-cultures of A549 and RAW264.7, only in the presence of serum. A: Western blot analyses of PRXV expression in co-cultures of the A549 and RAW 264.7 cells, stimulated with PAO1 without serum, actin used as control. No up-regulation of PRXV occurred in cells. Note, that the amount of RAW264.7 used alone, was equal to the amount of cells, added to the A549 cells (5:1 – A549:RAW). B: Expression of PRXV was up-regulated in the epithelial cells following contact with bacteria (PAO1) in the presence of serum and in co-culture with immune cells (RAW 264.7). Western blot analyses of PRXV expression in co-cultures. Immunostaining for actin used as control. C: Expression of PRXV is moderately up-regulated in the A549 cells by *P. aeruginosa *PAO1 and by co-culture with RAW 264.7, with and without bacterial inflammation. Quantitative photometric data from Western blot analyses performed in 3 separate cultures. * – *p *< 0.05.

**Figure 4:**
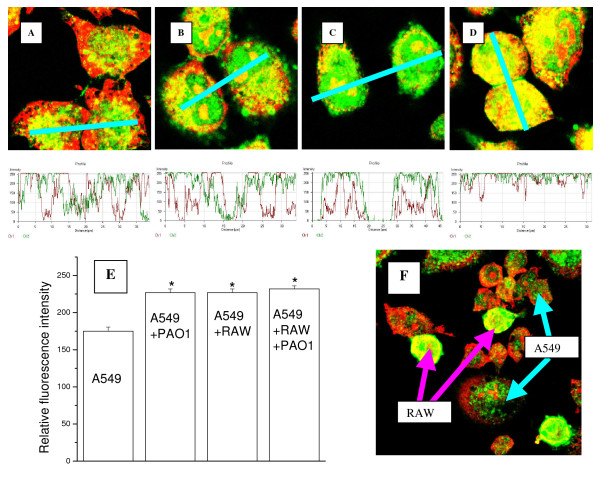
Co-culture of the alveolar epithelial cells with murine macrophages and stimulation by *P. aeruginosa *moderately upregulated PRXV expression, as determined by IHC and confocal microscopy. A-D: Typical confocal microscopy images of the A549 cultures, stained for PRXV (green fluorescence, FITC labeled secondary antibody) and co-stained with Propidium Iodine for DNA. A – control cultures (A549, no infection), B – cultures infected with PAO1; C – control co-cultures (A549 + RAW, no infection), D – cultures (A549+RAW264.7), infected with PAO1. At the bottom of each image, a diagram of the distribution of fluorescence intensity along the selected segment (red bar) is given. Green line is PRXV fluorescence intensity, red line is DNA fluorescence intensity. Original magnification × 100. E: MEAN data of relative fluorescence intensity for PRXV staining in co-cultures of the A549 and RAW264.7 cells. F: The RAW264.7 cells expressed higher amounts of PRXV than the A549 cells in co-cultures. Confocal microscopy images of co-cultures – both types of cells are indicated by labeled arrows. Staining for PRXV with FITC-labeled secondary antibody (green fluorescence). Co-staining – Propidium Iodine (red).

Experiments were first performed in the alveolar epithelial cell line A549, co-cultured with mouse macrophage cell line RAW264.7, both with and without the presence of serum. Western blot analyses demonstrated that co-culture of A549 with RAW264.7 and stimulation with PAO1 resulted in enhanced expression of PRXV only in the presence of serum, as shown in Figure 3. Results of quantitative IHC are shown in Figure 4. In the presence of serum, the addition of live *P. aeruginosa *modestly increased PRXV expression in A549 cultures, as well as in co-cultures with RAW264.7. *P. aeruginosa *bacteria itself were not positive for PRXV staining. The levels of PRXV mRNA did not change significantly in this system (data not shown). As can be seen from Figures 3 and 4, RAW264.7 expressed higher amounts of PRXV than the epithelial cells in culture, which is similar to our findings *in vivo*. However, small amounts of RAWs in the co-culture (5:1 ratio of epithelial cell/macrophages) did not significantly influence the overall expression of PRXV in the co-culture system, as epithelial cells were the predominant cell type.

**Figure 5:**
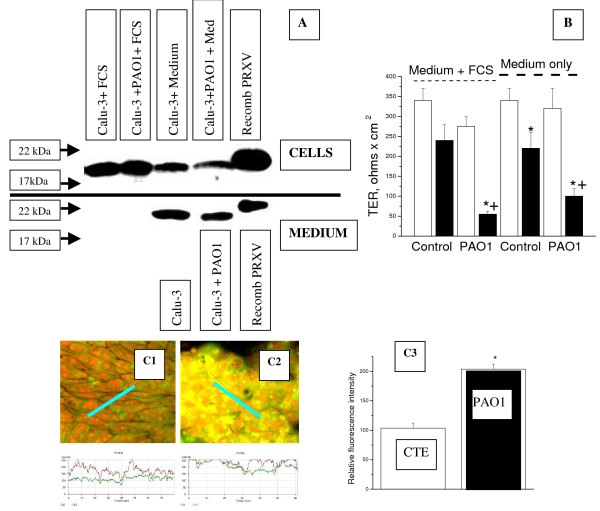
*P. aeruginosa *infection did not up-regulate expression of PRXV in the human bronchial epithelial cells Calu-3, as it did in the cow primary tracheal epithelial cell cultures. A: Western blot results of PRXV expression of the Calu-3 cell lysates (CELLS) and the cell-conditioned medium (MEDIUM). The Calu-3 cells were stimulated with PAO1 bacteria either in the presence of FCS or without it. PRXV was not upregulated in these cells, and its secretion in the medium was not changed. To confirm that PAO1 induced alterations in Calu-3 layers, TER of epithelial layers was measured. B: Summary results of TER following exposure of the Calu-3 epithelial cells to PAO1 with and without FCS. PAO1 induced a significant decrease in TER, which was more pronounced in the presence of FCS. Open bar – initial TER, closed bar – TER after a 4-hour exposure to medium or bacteria. In the "control" condition, cells were exposed only to the medium (or the medium with FCS) without bacteria. * – *p *< 0.05 compared to the initial value, ^+ ^– *p *< 0.05 compared to the control without bacteria, n = 6. C: *P. aeruginosa *infection up-regulated expression of PRXV protein in the primary cultures of the cow tracheal epithelial cells. C1-C2: Typical confocal microscopy images of the CTE cultures stained for PRXV (green fluorescence, FITC labeled secondary antibody) and co-stained with Propidium Iodine for DNA. C1 – control cultures (no infection), C2 – cultures infected with PAO1 for 12 hours. At the bottom of each image, a diagram of distribution of fluorescence intensity along the selected segment (blue bar) is given. Green line is PRXV fluorescence intensity, red line is DNA fluorescence intensity. Original magnification × 63. C3: MEAN data are given for fluorescence intensity in the control and PAO1-infected CTE cultures.

Using the Calu-3 bronchial epithelial cell line, which permits electrically resistant cell layers to be obtained, we measured TER, mRNA levels, and the expression of PRXV. We used TER as a measure of tight junctional electrical permeability, a characteristic of the epithelial phenotype. Following exposure to *P. aeruginosa*, the TER of these cells significantly decreased (*p *< 0.05), indicating that – in this model – addition of bacteria produced a considerable damaging effect on the epithelial cell layers (Figure 5). However, neither PRXV protein expression nor PRXV mRNA levels changed after exposure to PAO1; the mean relative PRXV protein expression following exposure to PAO1 was 106 ± 25% in the presence of serum and 76 ± 22% without serum as compared to baseline (Figure 5A).

Unlike Calu-3, the primary cultures of cow tracheal epithelium showed a pattern of increased PRXV expression after exposure to bacteria which was similar to the pattern shown by the A549 cells (Figure 5C).

**Figure 6:**
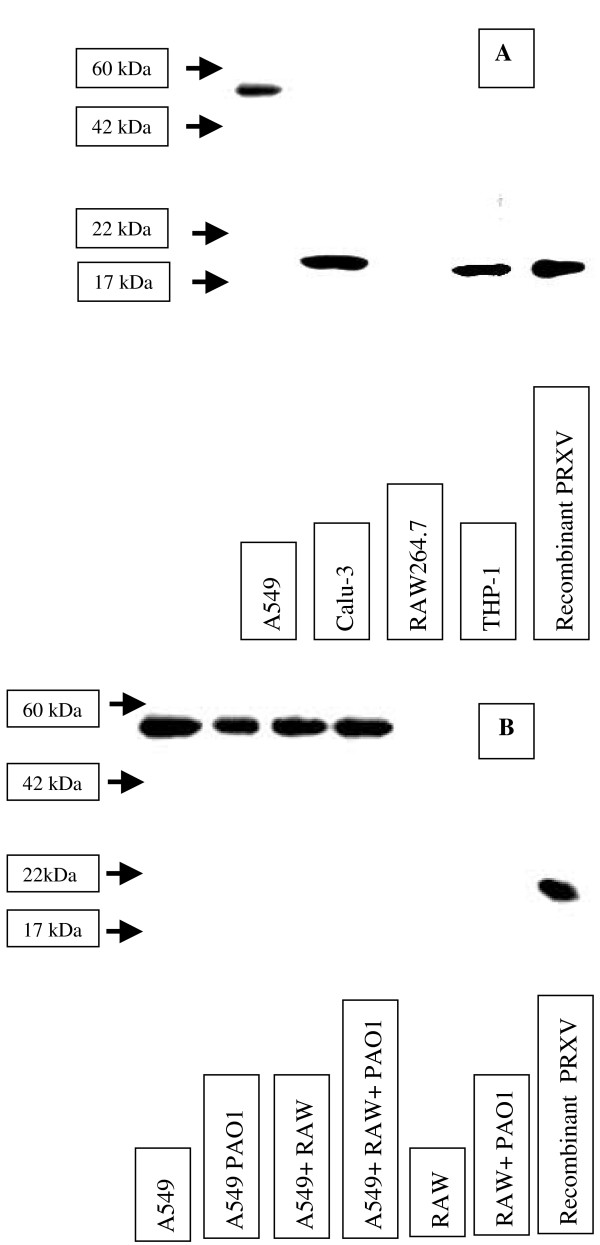
PRXV was secreted into the medium by the epithelial cells. Cell-conditioned medium from different cell cultures (A549, Calu-3, RAW264.7, THP-1) without FCS was analyzed by Western blot analyses for the presence of PRXV. Recombinant PRXV was used as the control. In the medium conditioned by the A549 cells, only a high molecular-weight form of PRXV (either polymer or possibly a glycosylated form) was present. The RAW 264.7 cells did not show appreciable amounts of PRXV secretion. B: Upon stimulation with PAO1 without FCS, secretion of PRXV into the medium by the A549 or RAW 264. 7 cells showed no change. Western blot analyses of cell-conditioned medium (without FCS) upon stimulation with PAO1. Western blot analyses of the medium with FCS provided substantial non-specific staining, precluding illustration.

Finally, using Western blot analyses of cell-conditioned medium with and without serum, we studied the presence of PRXV in the cell secretions of all cell lines that we used. Actin was used as a marker of intracellular non-diffusible proteins, and it was not found in the conditioned medium. Calu-3 and THP-1 secreted the monomeric form of PRXV into the medium (Figure 6A). THP-1, a human acute monocytic leukemia cell line was used here as positive control for inflammatory reaction. In the medium conditioned by the A549 cells, we observed only the PRXV form with approximately 60 kDa weight, which probably reflected polymer formation. We did not observe stimulation of secretion by exposure to PAO1 in the medium with serum (data not shown) or in the serum-free medium (68 ± 21% of control) (Figure 6B).

## Discussion

We investigated both *in vivo *and *in vitro *models of the lung bacterial inflammation. In mice, rats, and cultures of human airway epithelium cells, PRXV was abundantly expressed under non-inflammatory control conditions. In rats, neither the presence of endotoxins nor f-MLP-induced migration of leukocytes in the tracheal epithelium changed mRNA levels of PRXV; f-MLP slightly increased expression of PRXV protein in the tracheal epithelium. In mice, bacterial inflammation of the lung resulted in a massive influx of leukocytes, which were the source of the increased PRXV in the lung tissues. In primary airway cell culture (cow) and alveolar epithelial cells A549, or co-culture of the epithelial cells with murine macrophages RAW264.7, exposure to live bacteria mildly, yet significantly, increased expression of PRXV protein. Transcription of PRXV protein was not increased by exposure to bacteria in the A549 or Calu-3 cells. PRXV was secreted *in vitro *by both the epithelial and immune cells.

PRXV is a protein abundantly expressed under the baseline conditions in the airway epithelium, and these observations suggest that the major pathophysiological mechanism of its overall up-regulation in the lung during gram-negative bacterial inflammation is a shift in tissue cell populations due to migrating leukocytes. In the *in vitro *cultured airway epithelia, expression of PRXV protein was only moderately up-regulated in bacterial inflammation, while no transcriptional up-regulation was observed.

Our experimental finding that serum is required for the effect of PAO1 on up-regulation of PRXV may have several explanations. The most obvious is that recognition of bacteria by epithelial cells requires serum factors. Epithelial cells, unlike immune cells, do not possess receptors of innate immunity (Toll receptors and auxiliary proteins) in sufficient quantity. It is known, that epithelial and endothelial cells without the presence of immune cells are activated with bacterial products like lipopolysaccharide only in the presence serum. Generation of a response to bacterial products in non-immune cells occur only at a very high levels of bacterial product concentrations. We did not observe up-regulation of PRXV in co-culture of the epithelial and immune cells. Inflammatory reactions are complex even in this simplified *in vitro *model, with multiple loops of feedback regulation, both positive and negative. Likely, PAO1 caused activation of RAWs and possibly apoptosis of these cells. Activated RAWs release an array of pro-inflammatory cytokines, which might initiate apoptosis in epithelial cells and therefore decrease PRXV expression. The fate of RAWs co-cultured with the epithelial cells is difficult to estimate, but very likely RAWs did not have much survival advantage in the medium designed for epithelial cells.

Prior studies of PRX expression showed that PRXI, II, III, V and VI are highly over-expressed in the human lung cancer cells [21]. Allergic inflammation in response to ovalbumin induced overexpression of PRXI [22], which is also well known to be induced by hyperoxia [23]. Stimulation of the A549 cells and BEAS 2 B cells with hydrogen peroxide, menadione, tumor necrosis factor α, or transforming growth factor β did not result in significant changes of PRXV expression [18]. These *in vitro *results are in agreement with our data.

In studies of secreted PRXV, we observed only a 60 kDa band by Western blot analyses. Peroxiredoxins may form polymers in an oxidized state. It is unlikely that the band of interest was non-specific staining, simply because it was observed only after stimulation, but not in control non-stimulated cells and not in serum. Further investigations are needed to define the mechanisms of PRXV polymerization in extra-cellular fluids.

Some insights into possible mechanisms of PRXV gene regulation can be obtained by analysis of the PRXV gene structure. The PRXV gene is located on human chromosome 11q13, which is a region of genetic linkage for atopic hypersensitivity such as bronchial asthma. A 5' promoter region (4 kb upstream of the first exon) contains 3 potential binding sites (hypoxia-response element HRE, motifs ACGTG for hypoxia-inducible transcription factor HIF-1 and one potential antioxidant/electrophile response element (ARE/EpRE, motif TGACNNNGC). Additional ARE/EpRE is also present within the first intron, along with potential binding sites for transcription factor NF-kappa-B (motif GGRNAKTCCC) and Alu-associated retinoic acid-response element (RARE, motif AGGTSMNNAGWTCR). Therefore, in theory, transcription of this gene can be modulated in response to hypoxia, inflammation, and oxidative stress by intrinsic regulatory elements. It should be noted, however, that the functional activity of these potential transcription elements in the human PRXV promoter region has not been confirmed experimentally.

According to our data from the *in vivo *studies, PRXV protein is already abundantly up-regulated, and it is not up-regulated further by inflammation. One explanation is that the mechanisms regulating PRXV transcription cannot further increase expression in these cells, which are in constant direct contact with pathogens, antigens, and oxidants from the environment that are present in the bronchial tree *in vivo*.

Cells of the alveolar lining, however, are protected from these stimuli. It is unclear why we did not observe up-regulation of PRXV in the alveolar epithelium *in vivo *during bacterial inflammation of the airways, though we observed mild up-regulation in the A549 cells *in vitro*. Very likely, the model of lung bacterial inflammation that we used (instillation of bacteria into the airways) affected primarily the upper and conducting airways without causing massive inflammation in the alveolar spaces.

Adequate antioxidant protection in the lung is required for normal function, especially under conditions of oxidant stress caused by environmental factors [2]. Classic antioxidant enzymes of the lung cells that reduce hydrogen peroxide are catalase and glutathione peroxidase, while airway surface liquids and interstitial fluids are rich in superoxide dismutase and glutathione. Other hydrogen peroxide-reducing enzymes include thioredoxin-thioreductase, peroxiredoxins, and glutaredoxins [3]. Under physiologic conditions, superoxide dismutases and glutathione peroxidase are much more efficient than peroxiredixins in regulating the cell redox state. Under conditions of high oxidative stress, however, enzymes like thioredoxin may become physiologically important [1]. Hoshino and co-workers [24] showed that overexpression of thioredoxin or administration of its recombinant form protected mice against lung injury induced by pro-inflammatory cytokines and bleomycin. Adenovirus-mediated transfer of 1-cys peroxiredoxin gene was shown to protect mice from oxidative injury induced by exposure to oxygen [8]. PRXV has multiple functions: in addition to its antioxidant activity, PRXV is also a transcriptional co-repressor [13,14] and an inhibitor of p53-dependent apoptosis [10]. The anti-apoptotic activity of PRXV was demonstrated in tendon cells [9].

Our results showed that acute inflammation in the lung results in up-regulation of PRXV expression by different mechanisms. In the bronchial epithelium, we observed only a moderate rise in expression of this protein on a post-transcriptional level. Activated leukocytes that move to the lungs when inflammation occurs are a rich source of PRXV. However, this is likely to be a mechanism of leukocyte self-defense against self-induced oxidation rather than the mechanism of tissue protection. Defining the mechanism of PRXV regulation of expression in leukocytes during activation by mitogens was not the aim of present investigation and requires further study. Nonetheless, as PRXV is normally abundantly expressed in airways and as mechanisms of further PRXV up-regulation in the bronchial epithelium are limited, there is a basis for proposing therapeutic administration of this protein in recombinant form. Administration of PRXV in aerosol form may have significant therapeutic potential, especially in conditions where PRXV expression is down-regulated [15]. Inflammatory conditions are not the likely candidates for such an intervention, however, as there is no deficiency of PRXV in the airway epithelium during inflammation.

## Conclusion

*In vivo *and *in vitro *bacterial inflammation mildly up-regulates expression of PRXV protein in the airway epithelial cells. An increased influx of activated leukocytes to inflamed tissues serves as a source of enhanced expression of PRXV in the lung.

## Abbreviations

BSA – bovine serum albumin;

CSE – cigarette smoke extract;

CTE – cow tracheal epithelium;

FCS – fetal calf serum;

FITC – fluorescein isothiocyanate;

f-MLP – formyl-methionyl-leucyl-phenylalanine;

PBS – phosphate-buffered saline;

PRX – peroxiredoxin;

TER – transepithelial electrical resistance.

## Declaration of competing interests

The author(s) declare that they have no competing interests.

## Authors' contributions

RIK carried out experiments in cell cultures, Western blot analyses.

AVK carried out antibody development, experiments in cell cultures.

CL carried out Taq-man RT-PCR analyses.

VBS carried out animal experiments, IHC, study design, and drafted the manuscript.
